# Endovascular treatment of a giant arteria lusoria aneurysm in a young female with Marfan syndrome

**DOI:** 10.1093/bjrcr/uaaf005

**Published:** 2025-02-07

**Authors:** Sandra Fraund-Cremer, Rene Rusch, Anselm Uebing, Inga Voges, Philipp Schäfer

**Affiliations:** Clinic of Vascular and Endovascular Surgery, University Hospital of Schleswig-Holstein, Campus Kiel, D-24105 Kiel, Germany; Clinic of Vascular and Endovascular Surgery, University Hospital of Schleswig-Holstein, Campus Kiel, D-24105 Kiel, Germany; Department of Congenital and Pediatric Cardiology, University Hospital of Schleswig Holstein, Campus Kiel, D-24105 Kiel, Germany; German Center for Cardiovascular Research (DZHK), Partner site Hamburg/Lübeck/Kiel, Hamburg, Germany; Department of Congenital and Pediatric Cardiology, University Hospital of Schleswig Holstein, Campus Kiel, D-24105 Kiel, Germany; German Center for Cardiovascular Research (DZHK), Partner site Hamburg/Lübeck/Kiel, Hamburg, Germany; Clinic for Radiology and Neuroradiology, University Hospital of Schleswig-Holstein, Campus Kiel, D-24105 Kiel, Germany

**Keywords:** Marfan, aneurysm, ARSA, interventional therapy

## Abstract

Aneurysms of an aberrant right subclavian artery (ARSA) are rare but constitute a potentially lethal condition, especially with concomitant Marfan syndrome (MFS). A 27-year-old female with confirmed MFS presented with a relevant progression of a known aneurysm of an ARSA in MRI. The patient had undergone valve-sparing aortic root replacement (David procedure) 4 months prior. After interdisciplinary discussion, she underwent endovascular exclusion of the aneurysm using a combination of established vascular plugs and novel shape memory polymer embolization plugs to fill the large ARSA aneurysm volume. The shape memory polymer embolization plugs expand in the vessel to a porous scaffold, designed to support thrombus formation throughout its structure. The polymer is also radiolucent, which minimizes artefact and facilitates follow-up imaging. Development of a strategy for the treatment of ARSA aneurysms is challenging and different surgical, endovascular, and combined approaches have been published. Interdisciplinary discussion is crucial to minimize the overall risk and trauma. In our case of a young female and new mother, an endovascular approach was successfully and safely performed. The future need for surgery due to concomitant MFS is expected.

## Clinical presentation

**Figure 1.**
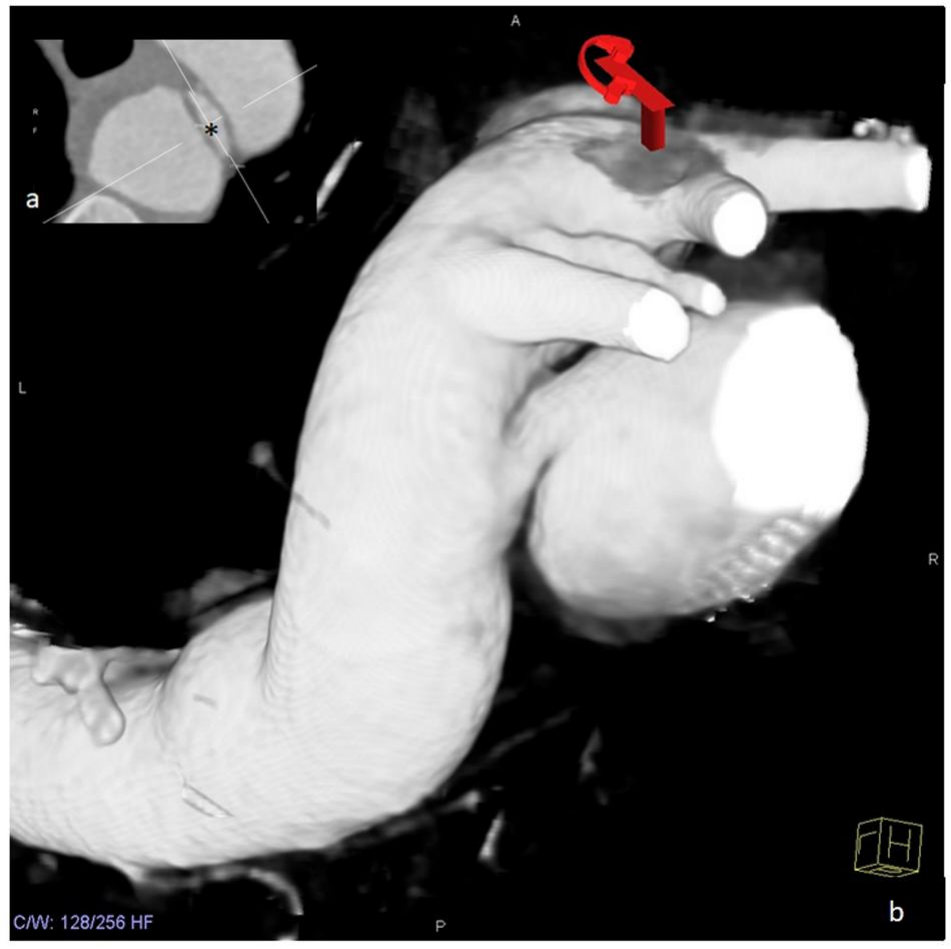
Multidetector CT (3D reconstruction) findings identified the ARSA aneurysm. Inset: an asterisk marks the proximal entrance of the aneurysm with a wide-open base.

A 27-year-old female presented with a progredient aberrant right subclavian artery (ARSA) aneurysm. She was diagnosed with Marfan syndrome (MFS) with a proven mutation (c.4786C), with regular surveillance imaging since 2007. In July 2014, dilatation of the aortic root (40 mm) and slight turbulence at the origin of the ARSA were evident on MRI, which were subsequently followed by echocardiographic examinations of the aortic root. In November 2021, the patient presented with pregnancy, and echocardiography showed an increased aortic root diameter and suspected aneurysmatic dilatation of the aortic arch. MRI showed a clearly enlarged aortic root (50 × 51 × 55 mm) and an ARSA aneurysm (24 × 32 × 48 mm). After intense interdisciplinary discussion, a valve-sparing aortic root replacement was performed at 22 weeks of gestation. The postoperative course was unremarkable for the mother and foetus, with a caesarean section at 35 weeks of gestation. MRI 1 month after the heart operation was unremarkable with respect to the ascending aorta and the ARSA diameter was unchanged. However, MRI/CT imaging at 4 months postpartum showed marked growth of the aneurysm (26 × 35 × 53mm, Figure 1).

**Figure 2.**
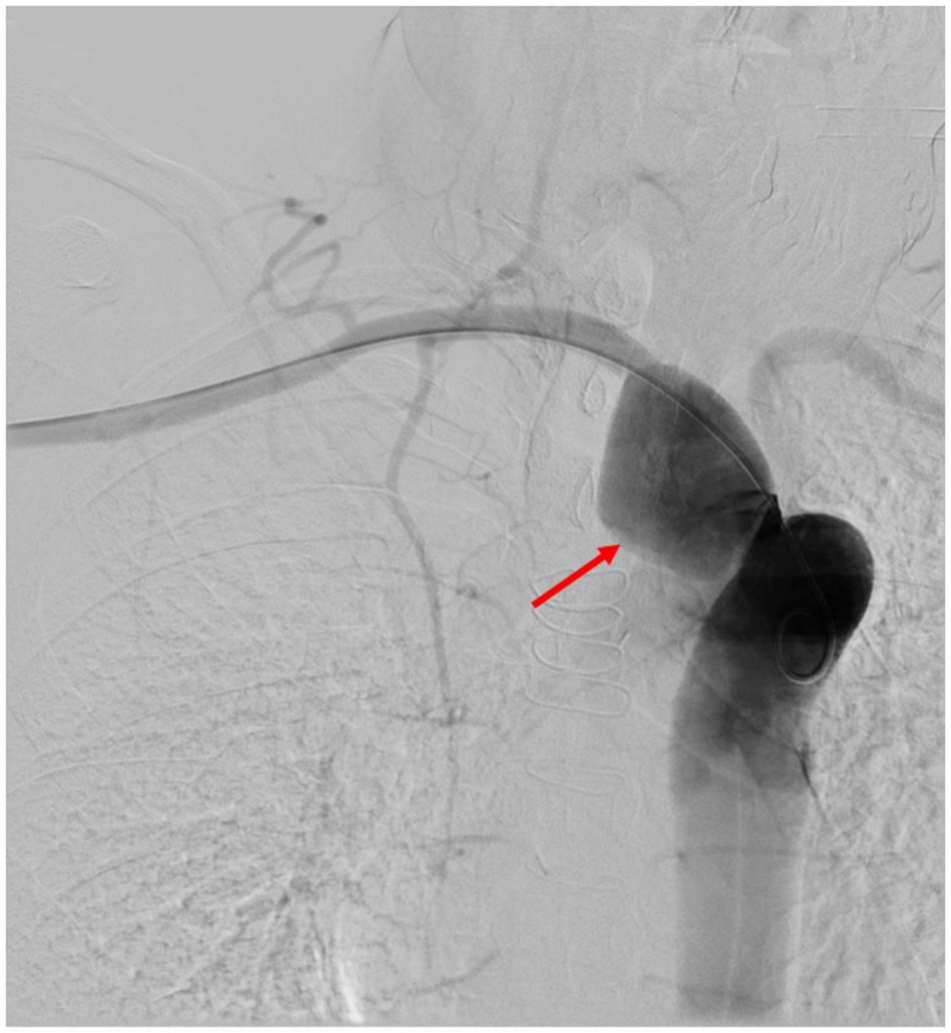
Selective intraarterial angiography of the right arm revealed the takeoff of the ARSA aneurysm (arrow) off the descending aorta at an acute and short angle, with just 5 mm length until the dilatation started.

The patient was generally in good condition (1.75 m, 87 kg). Cardiac examination revealed a slight diastolic heart murmur in the third intercostal space left, congruent with a slight aortic valve incompetence in echocardiography. Blood pressure and all pulses were normal. Contrast CT angiography (CTA) workup revealed a large ARSA aneurysm (∼55 mm diameter, 25 mL volume). The takeoff of the 19-mm diameter ARSA off the descending aorta was at an acute angle and short, with just 5-mm length until the dilatation started. Intravenous digital aortogram confirmed these findings (Figure 2), and revealed a significant predisposition to vasospasm.

## Treatment

**Figure 3.**
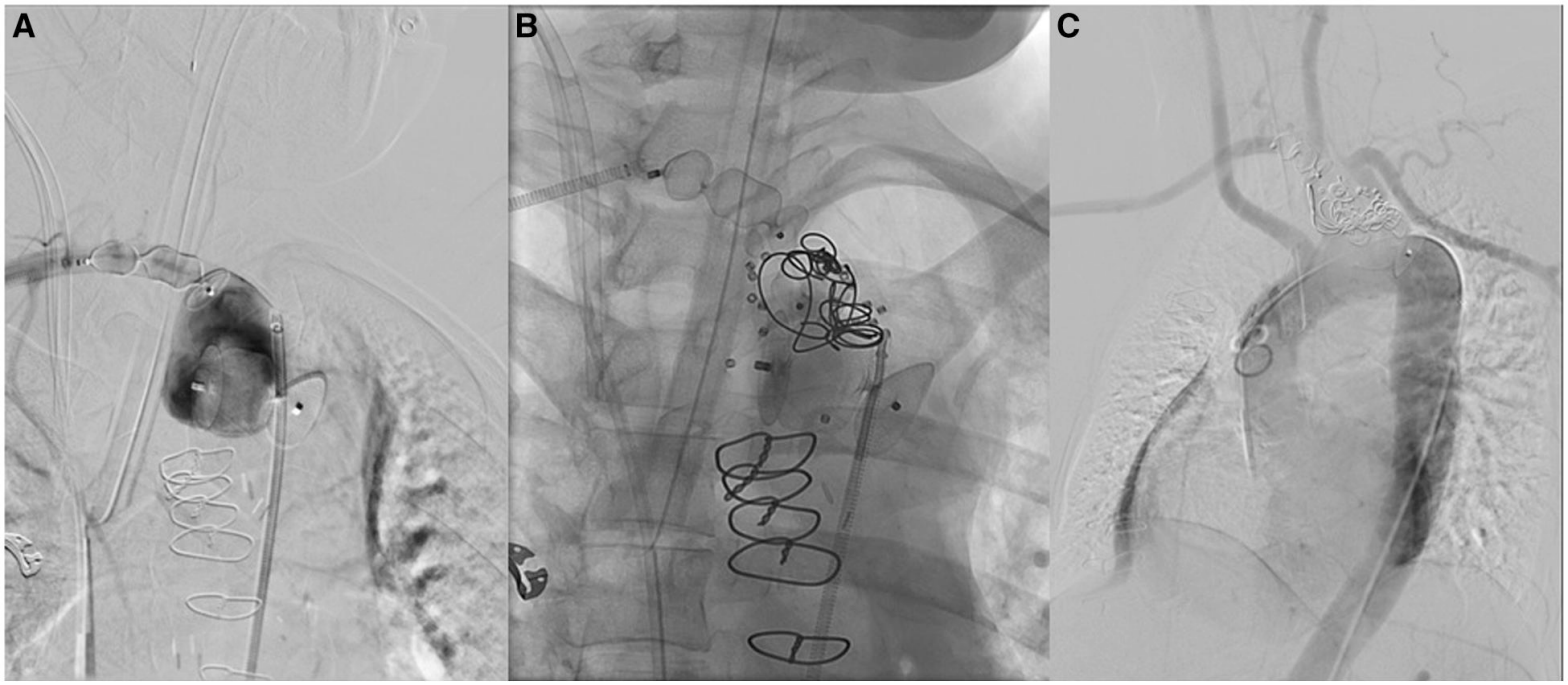
(A) The first step with proximal and distal plug embolization of the ARSA aneurysm with vascular plugs. (B) Aneurysm filling with coils and radiolucent shape memory polymer vascular plugs (visualized via small radiopaque markers). (C) Case completion angiography.

An interdisciplinary vascular board discussed open surgical and interventional methods. After detailed presentation of the options, the patient decided on an endovascular approach. As a pre-procedural precaution an MRI angiography of the supra-aortal and intracranial vessels (circle of Willis) was performed to ensure that the procedure can be carried out safely. Bilateral common femoral artery access was achieved, as well as retrograde direct puncture of the right brachial artery. Multi-segmental vasospasm complicated the access; angiography via a diagnostic microcatheter from the femoral artery via the ARSA revealed vasospasm in the distal brachial artery with strong collateralization perfusing the radial and ulnar arteries of the forearm. With transbrachial access in the true lumen established, a long 7-Fr sheath was placed into the descending aorta via the ARSA aneurysm. A 22 × 18-mm Amplatzer Vascular Plug II (AVPII) (Abbott, USA) was deployed into the ARSA, with the first lobe protruding into the aorta and the second and third lobes in the ARSA aneurysm. Then, a 12-mm diameter IMPEDE Embolization Plug (Shape Memory Medical, USA) was placed distal to the ARSA aneurysm, and proximal to the junction with the right vertebral artery. After several minutes, the shape memory polymer (SMP) plug had migrated distally, and was therefore snared (in the axillary artery) with blood aspiration to ensure removal of any polymer. After retrieval, there was no evidence of unintended embolization. With hindsight, the plug may have not been adequately sized for the vessel. A 14 × 10-mm AVPII was deployed instead; the first device dislodged proximally into the ARSA aneurysm, and therefore a second identical AVPII was deployed (Figure 3A). With balloon protection, a 6-Fr sheath was advanced into the ARSA aneurysm over the safety wire originally in place from the femoral approach. Then, 20 IMPEDE-FX Embolization Plugs (5 individual plugs and 3 × 5 plugs via the IMPEDE-FX RapidFill device; Shape Memory Medical, USA) were implanted into the ARSA aneurysm, in addition to 6 12-mm × 24-cm Nester Embolization Coils (Cook Medical, USA) (Figure 3B).

Completion angiography showed no discernable antegrade perfusion of the ARSA aneurysm, good reflux into the right vertebral artery, and good collateralized perfusion of the right arm (Figure 3C). CTA on postoperative day (POD) 1 showed complete embolization of the ARSA aneurysm, with faint residual contrast at the base. Postoperative duplex ultrasound examination confirmed occlusion of the right subclavian artery with retrograde flow in the right vertebral artery (Steal grade III) and sufficient collateralization of the posterior communicating artery. The neurological examination was unremarkable and the patient was discharged on POD 5.

## Outcome and follow-up

**Figure 4.**
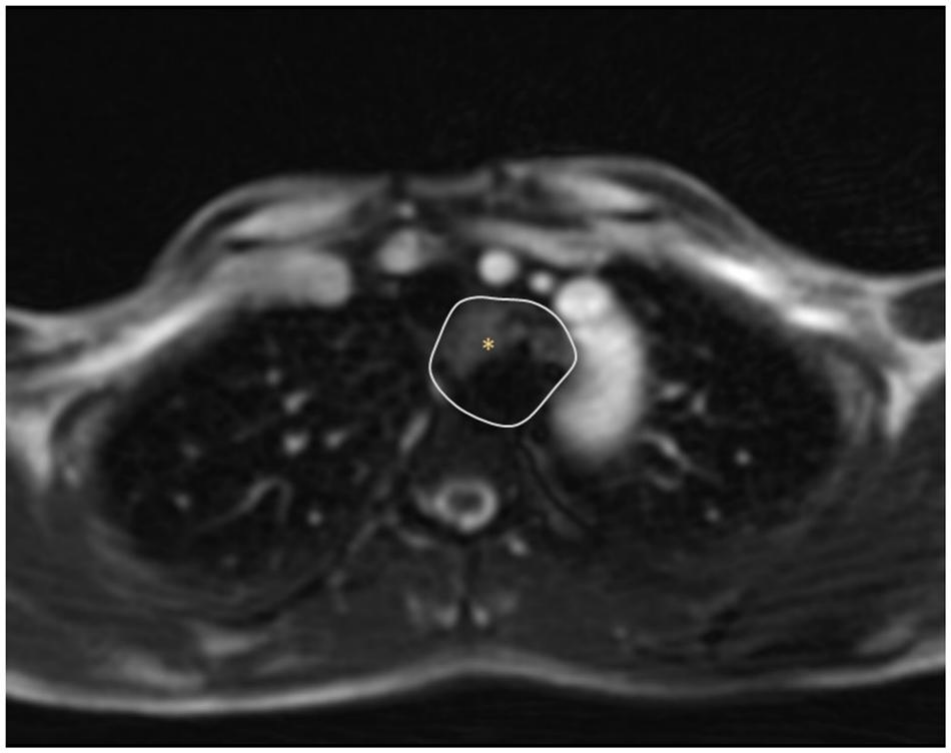
Magnetic resonance tomography at 26 months postprocedure showed the aortic arch with adequate occlusion of the ARSA aneurysm; the asterisk marks the thrombosed ARSA aneurysm.

The patient was last seen 26 months postprocedure at the department for adult congenital heart disease. The dizziness described postprocedure (when looking up/bending down/raising arms) was no longer an issue. MR tomography still showed adequate occlusion of the ARSA aneurysm after more than 2 years (Figure 4).

## Discussion

There are multiple reports on ARSA aneurysm treatment, from open surgical repair in 1-2 steps, through hybrid approaches, to completely endovascular treatment with coil embolization of the ARSA without any revascularization. Open surgical repair requires thoracotomy and/or combined access sites for revascularization. Mortality rates were 18%-25% in early reports,^1^^,^^2^ but this has more recently decreased to 3.5% (30-day).^3^

Hybrid approaches combine exclusion of the artery via endovascular embolization or stenting with surgical bypass. In 10 cases, bilateral revascularization/exclusion of the subclavian arteries enabled >20-mm proximal aortic fixation distal to the common carotid artery origins for the arch endograft.^4^ Distal occlusion was achieved via surgical ligation or endovascular embolization. Then, surgical revascularization via bilateral carotid-subclavian bypass or subclavian transposition occurred 1-3 days prior to deployment of a thoracic aortic endograft.

The use of completely endovascular approaches is increasing^3^; however, long-term outcomes are still unknown and complications include endoleaks and ipsilateral upper extremity claudication. Stent grafting is the treatment of choice for thoracic aortic aneurysms and dissection. However, endovascular stent grafting in patients with MFS and other connective tissue disorders is currently considered off-label and guidelines/expert consensus statements do not recommend endovascular repair.^5^

We had the special situation of a young mother with MFS, who had recently undergone a cardiovascular surgical procedure. The preprocedural plan was to use an AVPII to occlude the inflow and outflow of the aneurysm, and jail a delivery catheter in the sac to subsequently fill the large residual aneurysm volume with embolic material. Our decision to use SMP vascular plugs (along with coils) to fill the aneurysm after isolation of the aneurysm sac was based on the novel properties of the embolic material.^6^^,^^7^ The SMP in the IMPEDE-FX Embolization Plug self-expands in a vessel, resulting in a porous embolic scaffold designed to support thrombus formation, and the SMP has low radial force and was therefore not expected to exert great force on the aneurysm wall. Furthermore, it is radiolucent and we considered the minimal metal content of the device an advantage in a young patient. Finally, preclinical studies have shown SMPs stimulate the immune response without chronic inflammation and slowly bioabsorb.^8^

Nevertheless, it is important to discuss the dislodging of the SMP in a smaller vessel, either due to an incorrect size specification or due to inadequate deployment speed, which can lead to potentially dangerous embolization and the need for time-consuming retrieval manoeuvres.

We believe our case shows endovascular repair of ARSA aneurysms is achievable, with an excellent result to date. Longer-term follow-up will show the aneurysm progression in this patient with challenging comorbidities.

## Learning points

- There are multiple reports on aberrant right subclavian artery (ARSA) aneurysm treatment, from open surgical repair in 1-2 steps, through hybrid approaches, to completely endovascular treatment with coil embolization of the ARSA without any revascularization.
- The use of completely endovascular approaches is increasing in ARSA aneurysm treatment, however, complications include endoleaks and ipsilateral upper extremity claudication.
- Endovascular repair of ARSA aneurysms is achievable, with an excellent result even in Marfan syndrome patients. Long-term outcomes are still unknown.

## Informed consent

Written informed consent was obtained from the patient for publication of this case review, including accompanying images.

## References

[1] Kieffer E , Bahnini A, Koskas F. Aberrant subclavian artery: Surgical treatment in thirty-three adult patients. J Vasc Surg. 1994;19:100-109; discussion 110. 8301723 10.1016/s0741-5214(94)70125-3

[2] Kamiya H , Knobloch K, Lotz J, et al Surgical treatment of aberrant right subclavian artery (arteria lusoria) aneurysm using three different methods. Ann Thorac Surg. 2006;82:187-190. 16798212 10.1016/j.athoracsur.2006.02.080

[3] Loschi D , Santoro A, Rinaldi E, et al A systematic review of open, hybrid, and endovascular repair of aberrant subclavian artery and Kommerell's diverticulum treatment. J Vasc Surg. 2023;77:642-649. 35850164 10.1016/j.jvs.2022.07.010

[4] Wooster M , Back M, Sutzko D, et al A 10-year experience using a hybrid endovascular approach to treat aberrant subclavian arterial aneurysms. Ann Vasc Surg. 2018;46:60-64. 28479468 10.1016/j.avsg.2017.03.174

[5] Svensson LG , Kouchoukos NT, Miller DC, et al; Society of Thoracic Surgeons Endovascular Surgery Task Force. Expert consensus document on the treatment of descending thoracic aortic disease using endovascular stent-grafts. Ann Thorac Surg. 2008;85(1 Suppl):S1-S41. 18083364 10.1016/j.athoracsur.2007.10.099

[6] Morgan RA , Loftus I, Ratnam L, et al Clinical experience with a shape memory polymer peripheral vascular embolisation plug: a case series. CVIR Endovasc. 2021;4:29. 33687582 10.1186/s42155-021-00214-w PMC7943681

[7] Castellano D , Boghi A, Di Maggio L, Rapellino A, Savio D. Shape memory polymer ovarian vein embolisation in a patient with nickel allergy. CVIR Endovasc. 2021;4:25. 33655366 10.1186/s42155-021-00212-y PMC7925783

[8] Jessen SL , Friedemann MC, Ginn-Hedman AM, et al Microscopic assessment of healing and effectiveness of a foam-based peripheral occlusion device. ACS Biomater Sci Eng. 2020;6:2588-2599. 32715083 10.1021/acsbiomaterials.9b00895 PMC7380656

